# Insects as a Prospective Source of Biologically Active Molecules and Pharmaceuticals—Biochemical Properties and Cell Toxicity of *Tenebrio molitor* and *Zophobas morio* Cell-Free Larval Hemolymph

**DOI:** 10.3390/ijms25137491

**Published:** 2024-07-08

**Authors:** Teodora Knežić, Miloš Avramov, Vanja Tatić, Miloš Petrović, Ivana Gadjanski, Željko D. Popović

**Affiliations:** 1 Center for Biosystems, BioSense Institute, University of Novi Sad, 21000 Novi Sad, Serbia; teodora.knezic@biosense.rs; 2 Department of Biology and Ecology, Faculty of Sciences, University of Novi Sad, 21000 Novi Sad, Serbia; milos.avramov@dbe.uns.ac.rs (M.A.); vanja.tatic@dbe.uns.ac.rs (V.T.); 3 Department of Plant and Environmental Protection, Faculty of Agriculture, University of Novi Sad, 21000 Novi Sad, Serbia; milos.petrovic@polj.edu.rs

**Keywords:** insect hemolymph, yellow mealworm, superworm, biochemical properties, antioxidative status, cell toxicity, alternative proteins

## Abstract

Insects are of great interest as novel sources of alternative proteins and biologically active compounds, primarily anticancer agents. Protein-rich insect larval hemolymph is a prospective candidate for pharmaceutical and food industry-related research. In this study, selected biochemical properties and cell toxicity of larval hemolymph from two mealworm species, *Tenebrio molitor* and *Zophobas morio*, were analyzed. Total proteins and carbohydrates, antioxidant capacity, and the level of lipid peroxidation were determined. Human cancer (U-87) and normometabolic (MRC-5) cells were treated with different concentrations of larval hemolymph proteins, and the effects on cell viability were assayed 24, 48, and 72 h after treatments. *Z. morio* hemolymph was shown to be richer in total proteins, showing a higher antioxidant capacity and lipid peroxidation level than *T. molitor* hemolymph, which was richer in total carbohydrates. Cytotoxicity assays showed that *T. molitor* and *Z. morio* hemolymphs differently affect the viability of U-87 and MRC-5 cells in cell type-, dose-, and time-dependent manners. Hemolymph from both species was more cytotoxic to U-87 cells than to MRC-5 cells, which was particularly prominent after 48 h. Additionally, a more potent cytotoxic effect of *Z. morio* hemolymph was observed on both cell lines, likely due to its higher antioxidant capacity, compared to *T. molitor* hemolymph.

## 1. Introduction

In food science, there is widespread discussion regarding the integration of alternative proteins into new food products. The challenges of producing considerably more food for a growing global population with concurrent reduction in the environmental footprint of our agricultural systems must be addressed. With the world population projected to reach approximately 9.8 billion people by 2050, both the accessibility and affordability of alternative proteins are to be taken into account if they are to play a substantial role in addressing these challenges [1]. Showing great potential for future food systems, edible insects are considered an environmentally friendly choice as alternative sources of proteins. Their primary benefit is reflected in a good nutritional profile: high percentage of protein with high-quality amino acids, fatty acids (e.g., omega-3), fibers, vitamins (e.g., vitamin B12), and minerals such as calcium and iron [1,2], as well as the highly efficient conversion of ingested matter into biomass. Secondly, the production of insects for food and feed significantly reduces the environmental footprint by generating less greenhouse gasses and ammonia emissions, as well as by using considerably less water, energy, and land in comparison with conventional animal farming [3,4]. Additionally, many insects possess the potential for recycling agricultural waste products, which they can use as feeding substrates and transform them into nutritious food and feedstuff, which is then returned to the production cycle. Therefore, insects are ideal candidates for integration into the framework of the circular economy concept [5], while the consumption of insects positively contributes to both the environment and human health.

It is estimated that there are more than 2000 insect species used for human consumption, mainly in tropical countries [6,7]. However, according to the Food and Agriculture Organization (FAO) and Wageningen University & Research (WUR), insects can be considered a viable option for both food and feed, not only in tropical regions but in other global regions as well [8]. In recent years, the subject of the global exploitation of insects as food and feed has been the focus of exploration and study in a plethora of scientific publications [7,9,10,11].

Today, mealworms represent one of the more prevalent groups of insects that are used as alternative sources of protein worldwide. Although the yellow mealworm *Tenebrio molitor* and superworm *Zophobas morio* (Coleoptera: Tenebrionidae) are known as pests of secondary storage products such as grain, flour, and bran, and have therefore been considered unsuitable for human and animal consumption for a very long time [12,13], their notorious status began to change in the early 1980s. It was at that time that the National Aeronautics and Space Administration (NASA) announced that they were exploring the use of insects as an alternative protein source during their space missions [14]. Since then, mealworms have been one of the focal points of research towards discovering promising alternatives for conventional sources of proteins for both human and animal consumption [2,15,16,17,18,19,20,21]. Consequently, in 2022, *T. molitor* was approved by the European Food Safety Authority (EFSA) as a novel food, i.e., insect species safe for human consumption, which allowed for the mass upscaling of production in numerous European countries [22]. Contrary to its smaller relative, the superworm does not yet have an official edible status, but because of its high-quality nutritional profile and numerous beneficial properties that have been discovered, recent research suggests that, in the near future, species belonging to the genus *Zophobas* may also be accepted as novel food [20,23,24].

In addition to their nutritional qualities, insects are known to have other beneficial effects on human health. Recent publications, such as the one by Stull et al. (2018), have demonstrated that edible cricket powder supported the growth of the probiotic bacterium *Bifidobacterium animalis*, thereby improving human gut health and reducing systemic inflammation [25]. Also, de Carvalho et al. (2019) have shown that *T. molitor* flour has a potential prebiotic effect [26], while protein hydrolysates made from edible powders of *T. molitor* and *Acheta domesticus* (house cricket) have significant antioxidative power [27], potentially higher than fresh orange juice and olive oil [28,29]. Similarly, Zielińska et al. (2017) suggested that, together with other edible insect species, *Z. morio* larvae represent a valuable and unexploited source of proteins whose peptides have high antioxidative activity. The results obtained in this study also showed that selected edible insects, after in vitro digestion, have higher antioxidant activity compared to some protein hydrolysates obtained from plants or other animal products [30]. As for other mealworm species, it has been shown that peptides generated from a protein found in the lesser mealworm *Alphitobius diaperinus* represent dipeptidyl peptidase IV (DPP IV) inhibitors that play a role in glucose metabolism, and thus in the management of type 2 diabetes [31]. Although the research was conducted on animal model organisms, it was shown that *T. molitor*- and *Z. morio*-based full-fat meals, as functional feed additives, increased the growth performance of broiler chickens and changed the traits of their immune system [32], while defatted *Z. morio* larvae meals can lead to immunomodulation in the gilt-head seabream fish *Sparus aurata* [33].

When it comes to *T. molitor* and *Z. morio* specifically, different larval extracts of these insects have been shown to exhibit remarkable anticancer activity [24,34,35,36,37,38,39]. However, the effect of larval hemolymph, as the main depot of various biologically active micro- and macromolecules such as proteins, hormones, and other metabolites, has not been sufficiently examined on different normometabolic and cancer cells. Such research was recently carried out by Mahmoud et al. (2020), who showed that the larval hemolymph and fat body of the flesh fly *Sarcophaga argyrostoma* (Diptera: Sarcophagidae) had a cytotoxic effect on the MDA-MB-231 cell line, that is breast adenocarcinoma cells [40]. Also, hemolymph from the stinkbug *Aspongopus chinensis* Dallas (Hemiptera: Heteroptera: Pentatomidae) has been proven to have antiproliferative and antimetastatic effects on murine (4T1) and human (HCC1937) breast cancer cell lines [41]. Further, Elfar et al. (2023) tested the anticancer effects of hemolymph extracts from three bee species (*Apis mellifera*, *Chalicodoma siculum*, and *Xylocopa pubescens*) on human liver cancer (HepG2) and human cervical cancer (HeLa) cells, where all hemolymph extracts resulted in the inhibition of cell viability against the tested cancer cell lines in a dose-dependent manner [42]. Finally, the findings obtained in the most recent study conducted by Osman et al. (2024) concluded that hemolymph from the American cockroach *Periplaneta americana*, tested on Solid Ehrlich carcinoma-bearing mice, has remarkable anticancer effects [43].

Although there has been recent research devoted to the antimicrobial and anti-inflammatory activity of *Z. morio* hemolymph rich in antimicrobial peptides [44,45,46], there is a lack of studies not only on the anticancer activity of hemolymph from this species and its relative *T. molitor* but also regarding their effects on different normometabolic cell lines. The latter research would aid the case for *Z. morio* to be granted the status of edible insect, while furthering the potential of *T. molitor* as novel food.

Taken altogether, the potential of insects as novel sources of alternative proteins and biologically active compounds merits further exploration. In particular, there is a constant need for suitable replacements to synthetic antioxidants that are added to food as protective substances against free radicals, such as reactive oxygen species (ROS), in part due to strict regulations of their use. Natural compounds, potentially found in insect hemolymph, would therefore be more desirable substitutes for consumers to enhance the quality of food. Besides this aspect, insect hemolymph is a prospective candidate for the discovery of novel pharmaceuticals and anticancer agents.

For those reasons, in this study, selected biochemical parameters of *T. molitor* and *Z. morio* larval hemolymph, as well the effects of cell-free hemolymph from these insect species on cancer and normometabolic cell lines, were analyzed. Biochemical analyses include assaying total protein and carbohydrate content, as well as antioxidative capacity and levels of lipid peroxidation as indicators of oxidative stress. The effects of different concentrations of *T. molitor* and *Z. morio* hemolymph proteins on the viability of U-87 glioblastoma cells and MRC-5 lung fibroblasts were quantified with the MTT assay 24, 48, and 72 h after treatment.

## 2. Results

### 2.1. Biochemical Analyses

**Figure 1:**
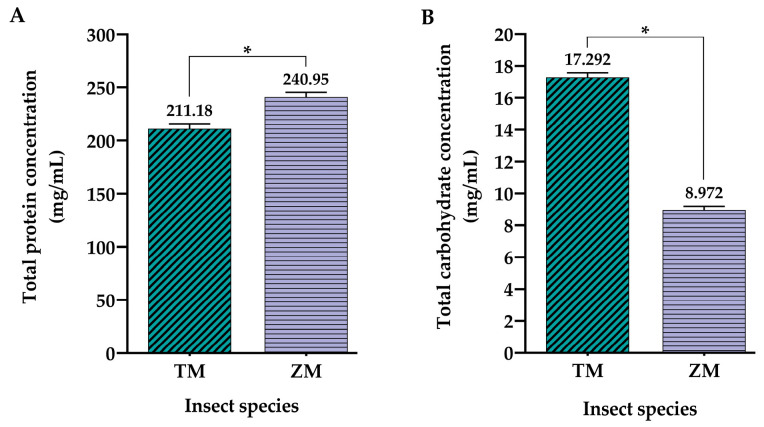
Total protein concentrations determined by Bradford assay (**A**) and total carbohydrate concentrations determined by anthrone reaction (**B**) from the larval hemolymph of selected insect species—*T. molitor* (TM) and *Z. morio* (ZM). Values are reported as the mean of triplicate measurements performed on hemolymph obtained from 100 larvae of each insect species, respectively, with the error bars representing the standard deviation of the measurements. Asterisks (*) above the bars denote statistically significant differences in the measured values of the biochemical parameters between the two different insect species, determined by one-way ANOVA followed by Tukey’s post hoc test for a significance level of *p* < 0.05.

The results obtained from the quantitative analysis of the biochemical properties of larval hemolymph from the two selected insect species have shown that protein content is higher in *Z. morio* samples in comparison to *T. molitor* samples (Figure 1A). On the other hand, hemolymph from *T. molitor* larvae contained nearly twice the concentration of total carbohydrates compared to *Z. morio* larval hemolymph (Figure 1B).

**Figure 2:**
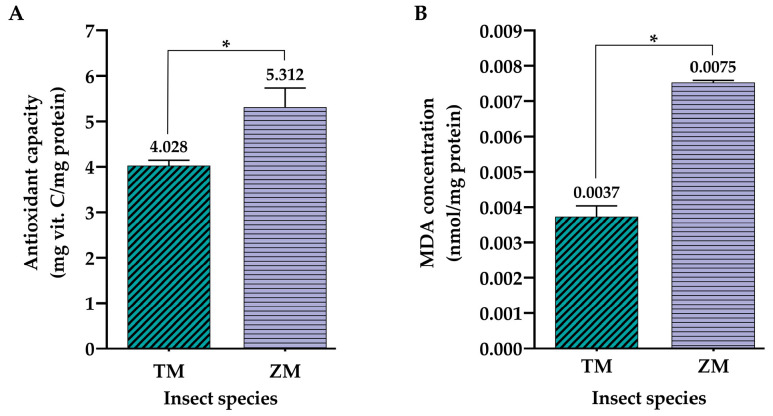
Antioxidative capacity of *T. molitor* (TM) and *Z. morio* (ZM) larval hemolymph measured by the antioxidative power of mg of vitamin C per mg of total protein, determined by FRAP assay (**A**), as well as lipid peroxidation levels measured in nmol of produced MDA per mg of protein, determined by MDA assay (**B**). Values are reported as the mean of triplicate measurements performed on hemolymph obtained from 100 larvae of each insect species, respectively, with the error bars representing the standard deviation of the measurements. Asterisks (*) above the bars denote statistically significant differences in the measured values of the biochemical parameters between the two different insect species, determined by one-way ANOVA followed by Tukey’s post hoc test for significance level of *p* < 0.05.

When it comes to the analysis of hemolymph antioxidative status, the results of ferric reducing antioxidant power (FRAP) and lipid peroxidation (MDA) assays have shown that the hemolymph of *Z. morio* has a higher antioxidative capacity, as well as level of lipid peroxidation, compared to *T. molitor* hemolymph. Using the antioxidative power of vitamin C as the standard, larval hemolymph from *Z. morio* was shown to have more than 30% higher antioxidative capacity than the hemolymph from *T. molitor* (Figure 2A). When it comes to the amount of malondialdehyde (MDA) produced, as a measure of lipid peroxidation levels, hemolymph from *Z. morio* was demonstrated to contain double the amount of MDA compared to its *T. molitor* counterpart (Figure 2B).

### 2.2. Cytotoxicity Assays

**Figure 3:**
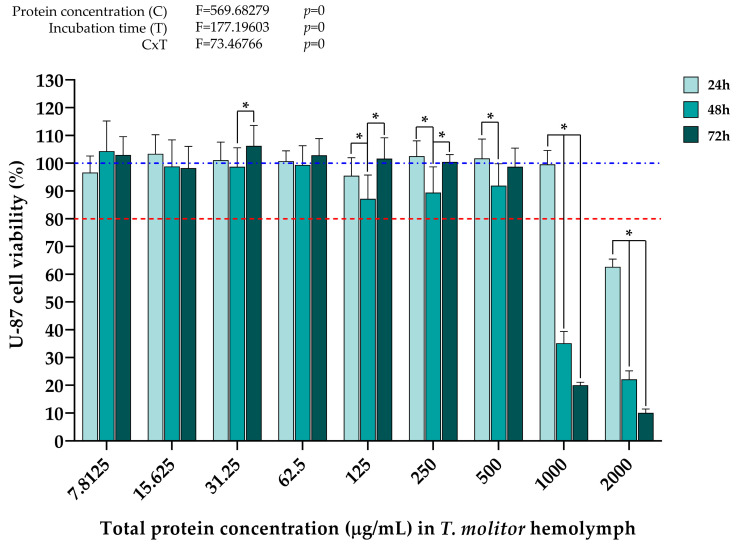
U-87 cell viability 24 h, 48 h, and 72 h after treatment with different *T. molitor* hemolymph total protein concentrations, determined by MTT assay. Error bars represent the standard deviation of three independent experiments, with four biological replicates each, for every measurement. The blue dashed line represents the control group taken as 100% cell viability. The red dotted line represents the viability cutoff of 80%, below which it is considered that the treatments are cytotoxic. Asterisks (*) above the bars denote statistically significant differences in measured cell viability between the corresponding bars linked with lines, determined by one-way ANOVA followed by Tukey’s post hoc test for significance level of *p* < 0.05. Interaction between protein concentration (C) and incubation time (T) for the whole experiment was analyzed by two-way ANOVA followed by Tukey’s post hoc test for significance level of *p* < 0.05 (top left).

The data in Figure 3 show that the highest concentration of *T. molitor* hemolymph proteins (2000 µg/mL) decreased cell viability below the 80% viability threshold already 24 h after treatment. A significant cytotoxic effect on U-87 cells also occurs 48 h after treatment with 1000 µg/mL of *T. molitor* hemolymph total proteins, with a nearly 70% reduction in cell viability compared to after 24 h. Cell viability was further negatively affected 72 h after treatment. Also, it was observed that hemolymph protein concentrations ranging from 125 µg/mL to 500 µg/mL exerted a slight negative effect on U-87 cell viability 48 h after treatment, whereby 72 h after treatment, a recovery of cell viability to the values recorded 24 h after treatment was noticed. Overall, hemolymph protein concentrations below 1000 µg/mL had no negative effect on U-87 cell viability at any time point. Additionally, according to the results of the two-way analysis of variance (two-way ANOVA), protein concentration and incubation time, as well as the interaction of these two factors, contributed to the decrease in U-87 cell viability.

**Figure 4:**
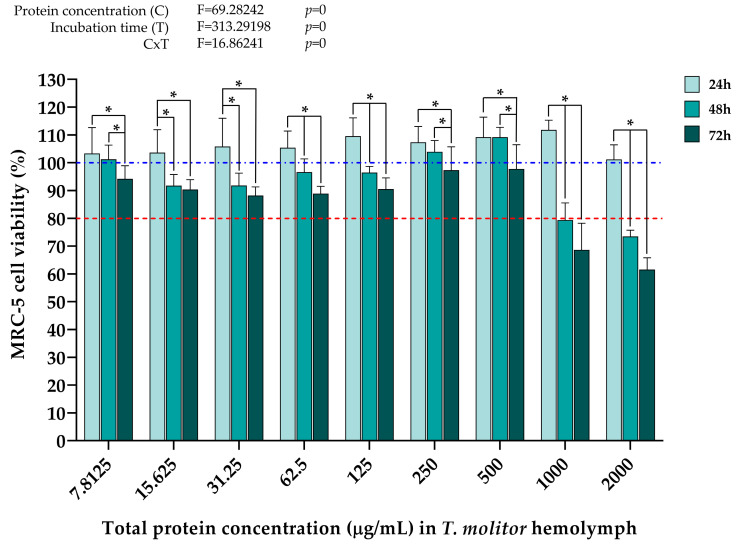
MRC-5 cell viability 24 h, 48 h, and 72 h after treatment with different *T. molitor* hemolymph total protein concentrations, determined by MTT assay. Error bars represent the standard deviation of three independent experiments, with four biological replicates each, for every measurement. The blue dashed line represents the control group taken as 100% cell viability. The red dotted line represents the viability cutoff of 80%, below which it is considered that the treatments are cytotoxic. Asterisks (*) above the bars denote statistically significant differences in measured cell viability between the corresponding bars linked with lines, determined by one-way ANOVA followed by Tukey’s post hoc test for significance level of *p* < 0.05. Interaction between protein concentration (C) and incubation time (T) for the whole experiment was analyzed by two-way ANOVA followed by Tukey’s post hoc test for significance level of *p* < 0.05 (top left).

The data in Figure 4 show the effect of different *T. molitor* hemolymph proteins on the viability of MRC-5 cells 24, 48, and 72 h after treatment. It can be seen that a slight cytotoxic effect first occurs 48 h after treatment with a total protein concentration of 1000 µg/mL. Interestingly, here, the treatment with 2000 µg/mL of hemolymph proteins did not have a negative effect on MRC-5 cell viability after 24 h, in contrast to the same treatment in the setup with U-87 cells. This concentration only started affecting MRC-5 cells 48 h after treatment, compared to 24 h for U-87 cells. Additionally, a general trend of a gradual decrease in MRC-5 cell viability over time was observed after all treatments. That being said, 500 µg/mL and below hemolymph protein concentrations did not bring cell viability below 80% even 72 h after treatment. As with the U-87 cell line, the results of the two-way ANOVA showed that protein concentration and incubation time, as well as the interaction of these two factors, had a significant effect on the decrease in MRC-5 cell viability after treatment with higher concentrations of *T. molitor* hemolymph proteins, as evidenced by the *p* values equaling 0, i.e., <0.05, for both analyzed factors and their interactions.

**Figure 5:**
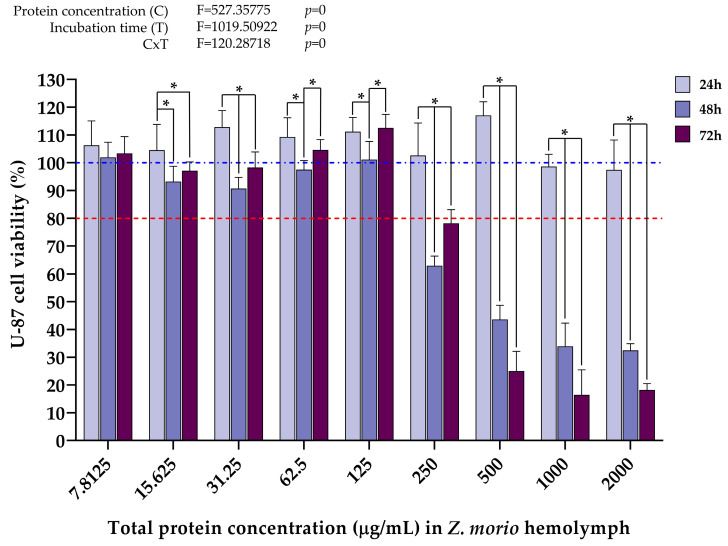
U-87 cell viability 24 h, 48 h, and 72 h after treatment with different *Z. morio* hemolymph total protein concentrations, determined by MTT assay. Error bars represent the standard deviation of three independent experiments, with four biological replicates each, for every measurement. The blue dashed line represents the control group taken as 100% cell viability. The red dotted line represents the viability cutoff of 80%, below which it is considered that the treatments are cytotoxic. Asterisks (*) above the bars denote statistically significant differences in measured cell viability between the corresponding bars linked with lines, determined by one-way ANOVA followed by Tukey’s post hoc test for significance level of *p* < 0.05. Interaction between protein concentration (C) and incubation time (T) for the whole experiment was analyzed by two-way ANOVA followed by Tukey’s post hoc test for significance level of *p* < 0.05 (top left).

When it comes to the effect of *Z. morio* hemolymph proteins on the viability of the U-87 cell line, the data in Figure 5 show that a significant cytotoxic effect first occurs 48 h after treatment with a total protein concentration of at least 250 µg/mL, where cell viability was reduced by approximately 40% compared to the values after 24 h. However, concentrations of 500 µg/mL and higher induce much stronger cytotoxic effects, with a reduction in cell viability of around 60% on average after 48 h. Interestingly, in contrast to the *T. molitor* samples, the protein concentration of 2000 µg/mL only started negatively affecting U-87 cell viability 48 h after treatment. Also, it was observed that hemolymph protein concentrations of *Z. morio* ranging from 15.625 µg/mL to 62.5 µg/mL exerted a slight negative effect on U-87 cell viability 48 h after treatment. Although less profound compared to the data obtained from *T. molitor* samples (Figure 3), a similar trend in cell viability recovery 72 h after treatment was observed. That being said, it can be seen that treatment with hemolymph protein concentrations up to and including 125 µg/mL did not negatively affect U-87 cell viability at any time point. As with *T. molitor*, the results of two-way ANOVA analyses showed that wherever a significant decrease in U-87 cell viability did occur, both protein concentration and incubation time, as well as the interaction between these two factors, were significant contributors to this effect.

**Figure 6:**
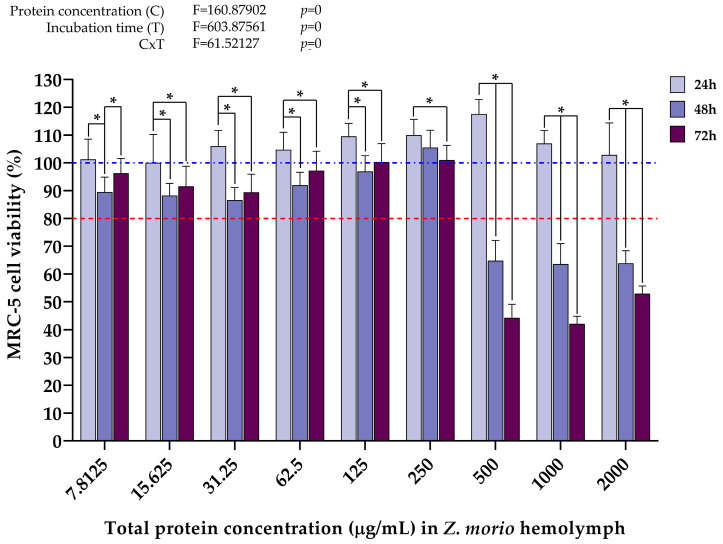
MRC-5 cell viability 24 h, 48 h, and 72 h after treatment with different *Z. morio* hemolymph total protein concentrations, determined by MTT assay. Error bars represent the standard deviation of three independent experiments, with four biological replicates each, for every measurement. The blue dashed line represents the control group taken as 100% cell viability. The red dotted line represents the viability cutoff of 80%, below which it is considered that the treatments are cytotoxic. Asterisks (*) above the bars denote statistically significant differences in measured cell viability between the corresponding bars linked with lines, determined by one-way ANOVA followed by Tukey’s post hoc test for significance level of *p* < 0.05. Interaction between protein concentration (C) and incubation time (T) for the whole experiment was analyzed by two-way ANOVA followed by Tukey’s post hoc test for significance level of *p* < 0.05 (top left).

The data in Figure 6 show that a significant cytotoxic effect on MRC-5 cells first occurs 48 h after treatment with at least 500 µg/mL of total proteins from the hemolymph of *Z. morio*, with around 60% reduction in cell viability compared to values after 24 h. Similar effects were induced by the highest concentrations 48 h after treatment. Also, it was observed that hemolymph total protein concentrations ranging from 7.8125 µg/mL to 125 µg/mL exerted a slight negative effect on MRC-5 cell viability 48 h after treatment, albeit the viability was still above the 80% cutoff. Although less profound compared to the results shown in Figure 4, the same trend in cell viability recovery 72 h after treatment is present. In general, only protein concentrations of 250 µg/mL and lower had no significant antiproliferative effect on MRC-5 cells, as opposed to *T. molitor* hemolymph proteins where no such effect was shown even at concentrations of 500 µg/mL. The results of the two-way ANOVA have shown the same trend as in previous cases, where protein concentration and incubation time, as well as the interaction of these two factors, had a significant effect on the measured decrease in MRC-5 cell viability after treatment with high hemolymph protein concentrations.

**Figure 7:**
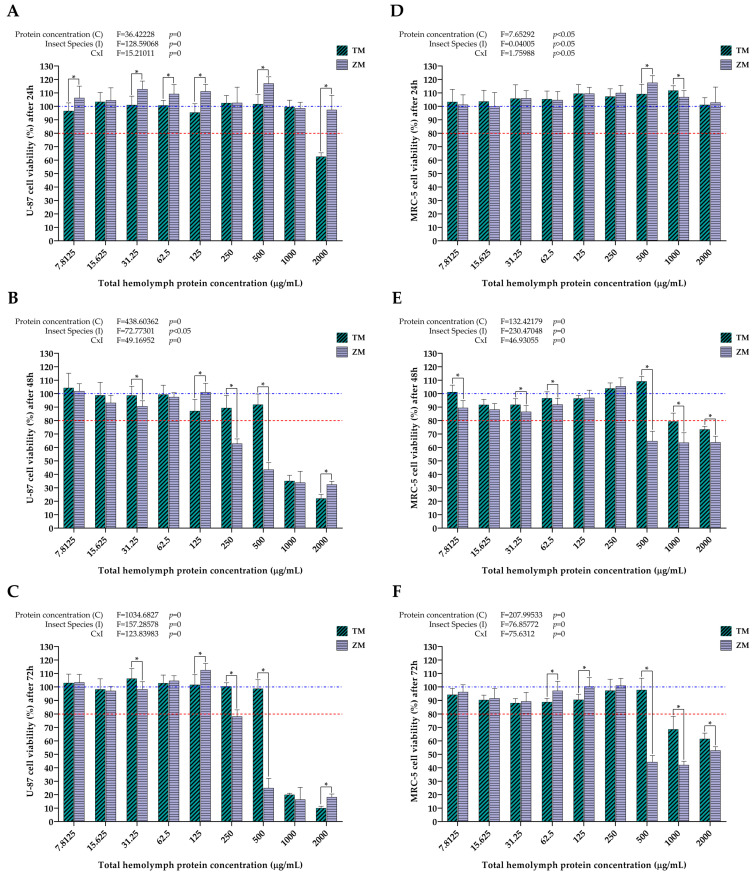
Comparisons of the effects of different *T. molitor* (TM) and *Z. morio* (ZM) hemolymph total protein concentrations on U-87 (24 h (**A**), 48 h (**B**), and 72 h (**C**) after treatment) and MRC-5 (24 h (**D**), 48 h (**E**), and 72 h (**F**) after treatment) cell viability, determined by MTT assay. Error bars represent the standard deviation of three independent experiments, with four biological replicates each, for every measurement. The blue dashed line represents the control group taken as 100% cell viability. The red dotted line represents the viability cutoff of 80%, below which it is considered that the treatments are cytotoxic. Asterisks (*) above the bars denote statistically significant differences in measured cell viability between the corresponding bars linked with lines, determined by one-way ANOVA followed by Tukey’s post hoc test for significance level of *p* < 0.05. Interaction between protein concentration (C) and insect species (I) for the whole experiment was analyzed by two-way ANOVA followed by Tukey’s post hoc test for significance level of *p* < 0.05 (top left).

Next, the two insect species were compared in regard to the after-treatment effects that the different protein concentrations have had on the viability of U-87 (Figure 7A–C) and MRC-5 cells (Figure 7D,E). Observing the results for U-87 cells (Figure 7A–C), it is noticeable that a pronounced cytotoxic effect was achieved already after 24 h with a concentration of 2000 µg/mL *T. molitor* hemolymph proteins, while all the other treatments did not have such an effect at the same time point (Figure 7A). At this concentration of *T. molitor* hemolymph proteins, the viability of U-87 cells was reduced by over 30% compared to the treatment with the same concentration of *Z. morio* hemolymph proteins. However, as time progressed, i.e., at 48 h after treatment, it was observed that higher concentrations of *Z. morio* hemolymph proteins (250 µg/mL and higher) exhibited more significant cytotoxicity on U-87 cells compared to the same concentrations of *T. molitor* hemolymph proteins (Figure 7B,C). This difference in cytotoxic effect was especially observed at a hemolymph protein concentration of 500 µg/mL, where the viability of U-87 cells was reduced by approximately 50% more 48 h after treatment (Figure 7B) and approximately 75% more 72 h after treatment with *Z. morio* hemolymph (Figure 7C), in comparison to treatment with *T. molitor* hemolymph. The exception is the protein concentration of 2000 µg/mL, where 72 h after treatment, a higher cytotoxic effect was still shown by *T. molitor* hemolymph proteins compared to those originating from *Z. morio*. Additionally, the results of the two-way ANOVA showed that factors such as protein concentration and insect species, as well as the interaction of these two factors, had a significant effect on the measured U-87 cell viability after all incubation time points.

When it comes to the results for MRC-5 cells, no cytotoxic effects were recorded for any protein concentration from both insect species 24 h after treatment (Figure 7D). However, as time progressed, it was observed that higher concentrations of *Z. morio* hemolymph proteins (500 µg/mL and higher) exhibited more significant cytotoxic effects on MRC-5 cells 48 h and 72 h after treatment, compared to the same concentrations of *T. molitor* hemolymph proteins (Figure 7E,F). In particular, 500 µg/mL of *Z. morio* hemolymph proteins reduced the viability of MRC-5 cells by around 50% and 60% more 48 h and 72 h after treatment, respectively, compared to the same concentrations of *T. molitor* hemolymph proteins. Additionally, the results of the two-way ANOVA showed that protein concentration and insect species, as well as the interaction of these two factors, had a significant effect on the measured MRC-5 cell viability 48 h and 72 h after treatment. On the other hand, in contrast to the results obtained for U-87 cells (Figure 7A, top left), protein concentration was the only factor that had a statistically significant effect on the measured MRC-5 cell viability 24 h after treatment, while the factor of insect species and its interaction with protein concentration was negligible (Figure 7D, top left).

## 3. Discussion

### 3.1. Biochemical Analyses

Following the biochemical analyses of cell-free larval hemolymph from the two insect species, the yellow mealworm *T. molitor* and superworm *Z. morio*, interesting findings were obtained regarding their composition and antioxidant properties. Although a relatively similar concentration of total hemolymph proteins was determined by the Bradford assay, with *Z. morio* hemolymph being slightly richer in proteins (Figure 1A), a significant difference was noted in the context of other biochemical parameters. Specifically, it was determined by the anthrone reaction that *T. molitor* hemolymph contained almost twice as much total carbohydrates as its *Z. morio* counterpart (Figure 1B). Additionally, FRAP and MDA assays have demonstrated the significantly higher antioxidant capacity of *Z. morio* hemolymph (Figure 2A), as well as the recorded level of lipid peroxidation reflected in the higher concentration of measured MDA (Figure 2B) compared to *T. molitor* hemolymph. It is possible that the increased lipid peroxidation, serving as an indicator of oxidative stress in the hemolymph of *Z. morio*, promoted a strong antioxidant response, thus accounting for the heightened antioxidant capacity observed for the hemolymph of *Z. morio* compared to that of *T. molitor*. Previous studies have shown that *Z. morio* hemolymph contains higher concentrations of total proteins known for their potential antioxidant properties [30,47,48,49], along with a significant presence of free amino acids, up to 200 mM in insect hemolymph [50]. Both of these characteristics contribute to the overall antioxidant potential of hemolymph in insects [51,52]. Similar findings were observed in the larval hemolymph of the housefly *Musca domestica*, wherein oxidative stress induced by an exogenous stressor triggered subsequent antioxidant responses. Notably, an increase in the level and activity of well-known protein-based antioxidants, the enzyme superoxide dismutase (SOD) and glutathione (GSH), was detected following the induction of oxidative stress [53]. Such changes in the protein profile were also reported in larvae of the silver-faced flesh fly *Sarcophaga argyrostoma*, where an induced state of oxidative stress was overcome by the increased production of new proteins, most likely antioxidants and repair proteins [54]. Also, the content of total proteins measured in the hemolymph samples of both insect species investigated in this study is consistent with the fact that cell-free insect hemolymph is rich in proteins, the most common of which are storage proteins, hexamerins composed of six ~80 kDa polypeptide subunits. The storage proteins are synthesized by the fat body and are particularly abundant in the last instar of an insect’s larval stage [50].

Regarding the measured total carbohydrate content, which was significantly lower in the hemolymph of *Z. morio*, the obtained values could also be related to active defense processes in this insect species caused by increased oxidative stress, i.e., lipid peroxidation. It has been shown that, in a state of stress, there is an increase in the level of MDA and a decrease in the larval energy reserves of various insect species such as *Galleria mellonella* [55,56] and *Spodoptera exigua* [57,58], implying a possible higher engagement of carbohydrates, such as glycogen, in the detoxification of the organism.

### 3.2. Cytotoxicity Assays

Following the cytotoxicity assays, significant differences were observed in the effect of different concentrations of *T. molitor* and *Z. morio* total hemolymph proteins on the viability of the U-87 and MRC-5 cell lines, as models of cancer and normometabolic cells, respectively. When it comes to the treatments with *T. molitor* hemolymph proteins, it can be seen that the antiproliferative effects on the U-87 cell line first occur 24 h after treatment with a concentration of 2000 µg/mL (Figure 3). On the other hand, in the MRC-5 cell line, significant cytotoxic effects were first observed when the cells were treated with 1000 µg/mL, but only 72 h after treatment (Figure 4). That being said, it should be noted that the viability of MRC-5 cells does show signs of decline 48 h after treatment with this protein concentration (Figure 4), but to a much lesser degree compared to the same treatment in the U-87 cell line (Figure 3). In that sense, the effects of *T. molitor* hemolymph proteins appear to be dependent on cell type and display potential anticancer properties. Similar findings were obtained in a recent study analyzing the effects of protein extracts from two aquatic insect species—*Helophorus aquaticus* and *Helophorus syriacus* (Coleoptera: Helophoridae) on the viability of human prostate cancer cells (PC-3), whereby the highest anticancer effect was demonstrated 48 h after treatment with samples containing 1000 μg/mL of insect protein extract. In addition, the examined protein extracts were shown to have high antioxidant capacity, which implies the existence of a link between the efficient anticancer potential and antioxidant properties of the tested biomolecules [59].

Cell treatments with *Z. morio* hemolymph proteins also seem to have similar cell type-dependent effects. Namely, a significant decline in the viability of U-87 cells occurs already 48 h after being treated with 250 µg/mL of hemolymph proteins from *Z. morio* (Figure 5), while it took twice the higher protein concentration (500 µg/mL) to begin inducing an antiproliferative effect in the MRC-5 cell line (Figure 6). The results obtained in this study and the very context of cell type-dependent effects that superworm hemolymph exhibits are supported by recent publications showing that extracts and anticancer peptides derived from *Z. morio* larvae had a significantly more negative effect on the viability of breast cancer cells (MCF-7) in comparison to normometabolic ones, human umbilical vein endothelial cells (HUVECs) [38] and Vero cells [35], respectively. Although a different insect species, it has also been shown that hemolymph from *S. argyrostoma* larvae had a stronger negative effect on the growth of breast adenocarcinoma cells (MDA-MB-231) compared to normometabolic Vero cells. In other words, it was demonstrated in vitro that the examined hemolymph exhibited anticancer activity [40]. Also, a protein hydrolysate from silkworm pupae (*Bombyx mori*) was shown to inhibit the growth of human gastric cancer cells (SGC-7901), while the proliferation of normometabolic model cell line, human embryonic kidney cells HEK293, was not affected [60]. Cell type-dependent effects of insect-derived agents were also observed for extracts of the American cockroach *Periplaneta americana*. The growth of human breast carcinoma cells (MCF-7) was significantly inhibited after treatment with the cockroach extracts, while the cytotoxic effect on normal lung fibroblasts (MRC-5) was considerably weaker [61]. In general, the higher antiproliferative effect of insect hemolymph on cancer cells, as opposed to normometabolic cells, could be due to the connection between the increased protein concentration in the insect hemolymph on one side and its antioxidant and anticancer activities on the other [42].

In the present study, the cell type-dependent effects of insect hemolymph treatments were also evidenced by a difference in the magnitude of the measured cytotoxicity, and not just in the concentrations at which cell viability is negatively affected. Significantly stronger cytotoxic effects of the treatments, measured in the decrease in cell viability, were recorded in the cancer cell line (U-87) compared to the normometabolic cells (MRC-5), regardless of insect species. At hemolymph protein concentrations that were shown to negatively affect the growth of U-87 cells 48 h after treatment (1000 μg/mL and 2000 μg/mL for *T. molitor* samples, 250 μg/mL and higher for *Z. morio* samples), the viability of U-87 cells was, on average, lower by ~45% and ~32% than that of MRC-5 cells after treatment with *T. molitor* (Figure 3 and Figure 4) and *Z. morio* hemolymph proteins (Figure 5 and Figure 6), respectively. Overall, these findings show similarities with the previously mentioned studies on the in vitro anticancer activity of insect hemolymph and insect-derived compounds. In that sense, the results of this investigation warrant further exploration in regard to the specific anticancer activity of the cell-free hemolymph extracts to better describe the recorded cytotoxic effects on the cancer cell line.

Apart from the cell type-dependent effects of the treatments with insect hemolymph, the results of the present study have also shown that the potency of the treatments was significantly different between the hemolymph extracts from the two analyzed insect species. When comparing the effects of different *T. molitor* and *Z. morio* hemolymph protein concentrations on the viability of both cell lines, it is evident that the latter species induces a significantly higher cytotoxic effect compared to the former in a predominantly dose- and time-dependent manner (Figure 7). With the exception of one treatment of U-87 cells, at a concentration of *T. molitor* hemolymph proteins at 2000 μg/mL, the viability of both cell lines was not affected by any of the treatments after 24 h (Figure 7A,D). At the next time point, 48 h after treatment, the differences in the cytotoxic effects between the two species become clear. In the case of U-87 cells, treatment with a concentration of *Z. morio* hemolymph proteins as low as 250 μg/mL reduced the viability of these cells below 80%. When it comes to *T. molitor*, only hemolymph protein concentrations of 1000 μg/mL and 2000 μg/mL were able to bring the viability of U-87 cells below 80% 48 h after treatment (Figure 7B). The observed effects further increased in intensity 72 h after the treatments, with stronger cytotoxic effects from treatments with *Z. morio* hemolymph proteins (Figure 7C). A similar trend was observed in the MRC-5 cell line, as well. There was no measurable cytoxocity from any of the treatments after 24 h, regardless of species (Figure 7D), with the first effects on cell viability becoming apparent 48 h after treatment with at least 1000 μg/mL of *T. molitor* hemolymph proteins and at least 500 μg/mL of *Z. morio* hemolymph proteins (Figure 7E). Similarly to U-87 cells, the cytotoxic effects of the treatments on the viability of MRC-5 cells further increased in intensity after 72 h, with the more potent effects being due to treatment with *Z. morio* hemolymph proteins (Figure 7F). Overall, the strongest cytotoxic effects were induced by hemolymph treatments after 48 h, when the sharpest reductions in cell viability of both cell lines were measured by the cytotoxicity assay. These observed differences might be due to size differences in the larvae, with *Z. morio* larvae weighing, on average, around 750 mg [62], while *T. molitor* larvae are only around 100 mg in weight [63]. In that sense, *Z. morio* larvae should be more robust, have a higher rate of basal metabolisms, and, subsequently, increase measurable oxidative stress and production of antioxidants. To reiterate, in the present study, it was shown that *Z. morio* larval hemolymph had a higher concentration of total proteins, higher antioxidant capacity, elevated levels of lipid peroxidation, and less total carbohydrate content than the hemolymph from *T. molitor* larvae (Figure 1 and Figure 2). As such, considering the connection between these biochemical parameters and the anticancer/antioxidative activities of insect hemolymph [30,42,47,48,49,51,52,55,56,57,58,59], the observed stronger cytotoxic effects of *Z. morio* hemolymph on both cell lines, compared to the hemolymph from *T. molitor*, are to be expected.

Lastly, it should be taken into consideration that the findings of this study were obtained from analyses where freshly extracted cell-free hemolymph was used. It is common practice to prepare samples in advance and freeze them until they are needed. However, the freezing–thawing process can potentially affect the biological properties of the samples that are meant to be analyzed and quantified, thus skewing the results. As indicated by a preliminary study, for example, treating normometabolic fibroblasts with previously frozen hemolymph from *T. molitor* and *Z. morio* larvae did not elicit cytotoxic effects [64], as opposed to the results from the present study when fresh hemolymph was used for cytotoxicity assays. The differences in the findings could be due to the sensitivity and loss of biological potency of hemolymph components that are responsible for the observed cytotoxic effects caused by the freezing and subsequent thawing of hemolymph samples.

## 4. Materials and Methods

### 4.1. Insect Rearing

Populations of *Tenebrio molitor* (yellow mealworm) and *Zophobas morio* (superworm) were provided by the Entomology Laboratory of the Department of Environmental and Plant Protection at the Faculty of Agriculture, University of Novi Sad, Serbia.

#### 4.1.1. Population of *Tenebrio molitor*

Yellow mealworm colonies were maintained in an incubator (Witeg GC-450, Wertheim, Germany) under controlled conditions in 12 L plastic containers (20 × 40 × 15 cm). The insects were raised on a food substrate mixture containing barley bran, wheat germ, and oat germs (4:2:1 by volume). Apple slices were added as an additional moisture source for the insects. As mentioned, the rearing was performed in an incubator with the following microclimate setup: temperature: 27 ± 1 °C; photoperiod: 0 h light–24 h dark; relative humidity: 55%.

During the rearing, the consumed food was sieved out and additional quantities were added to ensure the availability of fresh food for the insects. With such a diet, the life cycle of larvae usually lasts 90 to 95 days, after which they enter the pupation process. Larvae at the 15th or 16th larval stage (85 to 90 days old) were used for the purposes of the experiment.

#### 4.1.2. Population of *Zophobas morio*

The superworm colony was maintained with intermittent introductions of new adults in order to minimize inbreeding during rearing. The rearing process was divided into two stages. In the first stage, adults were placed in containers (40 × 60 × 15 cm) with a feeding substrate made up of wheat bran (95%) and dried beer yeast (5%) to copulate and lay eggs for 10 to 12 days. After this period, both dead and live adults were removed, and the substrate with eggs was transferred onto 40 × 60 × 8 cm trays. The trays were placed in an incubator with the following microclimate setup: temperature: 29 ± 1 °C; photoperiod: 0 h light–24 h dark; relative humidity: 55%.

During the rearing process, apple or carrot pieces were added in order to provide moisture to larvae and keep cannibalism at a minimum. Larvae at the 16th to 18th larval stage (105 to 110 days old) were used for the purposes of the experiment.

### 4.2. Hemolymph Extraction and Total Protein Assay

Prior to hemolymph extraction, *T. molitor* and *Z. morio* larvae were first anesthetized by placing them in containers with ethyl acetate and holding them at 4 °C. Afterwards, their first pair of legs was cut off with dissection scissors and hemolymph was collected in chilled sterile microcentrifuge tubes. One hundred (100) larvae of both insect species were used in order to collect a sufficient amount of hemolymph needed to determine the biochemical properties of the hemolymph, as well as for cell toxicity assays—around 1.5 mL and 3 mL of *T. molitor* and *Z. morio* larval hemolymph, respectively. Larvae of *T. molitor* yielded around 15 µL of hemolymph per insect on average, while *Z. morio* larvae yielded around 30 µL of hemolymph per insect on average. Following collection, hemocytes were removed from the hemolymph by centrifuging the samples for 5 min at 12,000× *g* and 4 °C using a microcentrifuge (Eppendorf 5415R, Hamburg, Germany), followed by the transfer of the supernatant to chilled sterile microtubes. It is necessary to emphasize that freshly extracted cell-free hemolymph was used for all downstream analyses in order to avoid potential changes in hemolymph properties due to the freezing–thawing process.

Total hemolymph proteins were determined using the Pierce™ Bradford Protein Assay Kit (Thermo Fisher Scientific, Waltham, MA, USA) following the manufacturer’s instructions. The provided bovine serum albumin (BSA) was used to prepare the following protein standard dilution series [mg/mL]: 0.125, 0.250, 0.500, 0.750, 1.000, 1.500, 2.000. Absorbance was measured at a wavelength of 595 nm using a spectrophotometer (Multiskan GO, Thermo Fisher Scientific, Waltham, MA, USA) and SkanIt version 5.0 software (Thermo Fisher Scientific, Waltham, MA, USA), after which a standard curve was plotted in Excel 2021 (Microsoft Corporation, Redmond, WA, USA). All measurements were carried out in triplicate.

### 4.3. Biochemical Properties

#### 4.3.1. Quantitative Determination of Total Carbohydrates by Anthrone Reaction

The carbohydrate content of insect hemolymph was quantitatively determined by anthrone reaction, in which hydroxymethylfurfural (HMF), formed by the hydrolysis of complex and subsequent dehydration of simpler carbohydrates with sulfuric acid (ccH_2_SO_4_), creates a blue–green complex with anthrone.

The anthrone reagent used in this experiment was prepared as follows: 360 mL of ccH_2_SO_4_, and then 250 mg of anthrone and 5 g of thiourea, were carefully added to 140 mL of distilled water (dH_2_O), after which the mixture was heated to 80–90 °C in a water bath (Precisterm^®^, JP Selecta™, Abrera, Catalonia, Spain) until everything dissolved. The following concentrations of glucose solution were used as standards [mg/mL]: 0.313, 0.625, 1.250, 2.5, 5, 10, 20, while dH_2_O was used as a blank. The following protocol was used: 0.025 mL of sample/standard and 2.5 mL of anthrone reagent were added to individual test tubes, and they were incubated for 15 min at 100 °C in a water bath and then cooled down to room temperature. Absorbance was measured at a wavelength of 620 nm using a spectrophotometer (Nicolet Evolution 100, Thermo Fisher Scientific, Waltham, MA, USA), after which a standard curve was made. Given that the glucose solution was used as a standard, a factor of 0.93 was applied when converting glucose to total carbohydrates during the final calculation. All measurements were performed in triplicate.

#### 4.3.2. Ferric Reducing Antioxidant Power (FRAP) Assay

The total antioxidant power of insect hemolymph was evaluated using a modified ferric reducing antioxidant power (FRAP) assay. The method is based on the reduction of iron (III) to iron (II) in an acidic environment. A blue complex of iron–tripyridyl triazine (Fe-TPTZ) is formed, and its maximum absorption can be measured at a wavelength of 593 nm. The antioxidative power was expressed as a value compared to the antioxidative capacity of vitamin C, which was used as a standard.

The working solution was made by mixing 300 mM acetate buffer pH 3.6, 20 mM FeCl_3_, and 10 mM TPTZ in a 10:1:1 ratio. The solution was prepared immediately prior to use and kept in the dark to prevent degradation by light exposure.

The standard, 10 mg/mL vitamin C, was made as a stock solution and subsequently diluted to 1 mg/mL and 0.1 mg/mL. The standard curve was made using the following amounts of vitamin C per well: 4 µg, 2 µg, 1 µg, 0.75 µg, 0.5 µg, 0.2 µg, 0.1 µg, and a blank with only dH_2_O.

The assay was conducted in a 96-well plate using 10 µL of hemolymph sample or vitamin C standard and 250 µL of the working solution. The plate was incubated at room temperature in the dark for 30 min, after which the absorbance was measured at a wavelength of 593 nm using a Multiskan GO spectrophotometer and SkanIt version 5.0 software. All measurements were performed in triplicate.

The antioxidative capacity for each sample is expressed as an equivalent of vitamin C using the following formula:

$$\mathrm{equivalent}\;\mathrm{of}\;\mathrm{vit}.\;C=\frac{\mathrm{FRAP}\;}{C_{\mathrm{protein}}\times V_{\mathrm{sample}}}[\frac{mg\;vitamin\;C}{mg\;protein}]$$

#### 4.3.3. Lipid Peroxidation (MDA) Assay

Lipid peroxidation in the hemolymph samples was determined using the MDA assay [65], where the concentration of malondialdehyde (MDA) is used to calculate the level of peroxidation in the samples. Malondialdehyde is formed during reactive oxygen species (ROS)-induced peroxidation of polyunsaturated fatty acids found in membranes. The assay is based on the reaction of thiobarbiturate with MDA, which forms a colored complex with a maximum absorbance at a wavelength of 530 nm.

The working solution for the MDA assay contains 15% trichloroacetic acid (TCA), 0.375% thiobarbituric acid (TBA), and 0.25 M HCl prepared in dH_2_O. Malondialdehyde was used as a standard in the following concentrations: 25 mM, 10 mM, 5 mM, 2 mM, 1 mM, 0.5 mM, and dH_2_O as a blank.

Assay reactions were prepared in 2 mL microtubes by mixing 200 µL of the hemolymph sample with 1 mL of the working solution and filling up the microtubes to 1.5 mL with dH_2_O. For the MDA standards, 500 µL of the standard solution was mixed with 1 mL of the working solution. Microtubes were sealed with parafilm and tape to prevent their caps from opening due to incubation. The mixtures were vortexed and heated to 95 °C in a water bath for 30 min to ensure protein denaturation. Afterwards, the mixtures were cooled down to room temperature and centrifuged at 10,000× *g* for 10 min in a microcentrifuge. Then, 250 µL of the supernatant was transferred to a 96-well plate where absorbance was measured at a wavelength of 530 nm using a Multiskan GO spectrophotometer and SkanIt version 5.0 software. All measurements were performed in triplicate.

Lipid peroxidation of each sample was expressed as nmol of MDA per mg of protein using the following equations:

$$X\;\mathrm{value}=\mathrm{MDA}\;\left[\frac{µ\mathrm{mol}}{L}\right]\times V_{\mathrm{solution}}\left[L\right];\;\mathrm{unit}:\left[\frac{µ\mathrm{mol}}{well}\right]$$

$$\mathrm{Lipid}\;\mathrm{peroxidation}=\frac{X\;\mathrm{value}[\frac{µmol}{well}]}{C_{\mathrm{protein}}\;[\frac{mg}{mL}]\times V_{\mathrm{sample}\;}\left[\mathrm{mL}\right]}$$

### 4.4. Cell Toxicity Assays

#### 4.4.1. U-87 and MRC-5 Cell Cultures

In this study, U-87 human glioblastoma cells (89081402; ECACC, Porton Down, Wiltshire, England) were used, as a model of cancer cells, as well as human lung fibroblasts—MRC-5 cells (CCL-171™; ATCC^®^, Manassas, VA, USA), which were used as a model of normometabolic adherent cells.

Before seeding for the experiment, U-87 and MRC-5 cells were grown in T-25 cell culture flasks (BioLite, Thermo Fisher Scientific, Waltham, MA, USA) in Eagle’s minimum essential medium (EMEM) (ATCC^®^, Manassas, VA, USA) containing Earle’s balanced salt solution, non-essential amino acids, and 2 mM L-glutamine, and in Dulbecco’s modified Eagle’s medium (DMEM) (Sigma-Aldrich, St. Louis, MO, USA) containing 4500 mg/L of glucose and 2 mM L-glutamine, respectively. Both cell culture media were supplemented with 10% fetal bovine serum (FBS) (Sigma-Aldrich, St. Louis, MO, USA) and a 1% penicillin–streptomycin solution (Pen-Strep) (Sigma-Aldrich, St. Louis, MO, USA). The U-87 and MRC-5 cells were seeded at a concentration of 0.5 × 10^6^ cells and 0.7 × 10^6^ cells, respectively, and incubated at 37 °C and 5% CO_2_ in a humified CO_2_ incubator (ICO50, MEMMERT, Schwabach, Germany). The cells were passaged twice per week and replenished with fresh cell culture media as needed.

For the experimental treatments, U-87 and MRC-5 cells were seeded in 96-well flat bottom TC-treated microplates (BIOFIL^®^, Guangzhou, China) at a concentration of 0.005 × 10^6^ cells/well and 0.02 × 10^6^ cells/well, respectively, in a total volume of 200 µL/well. The plates were incubated for 24 h at 37 °C and 5% CO_2_ in a humified CO_2_ incubator prior to the treatments with insect hemolymph.

#### 4.4.2. Treatment of U-87 and MRC-5 Cells with Different Total Hemolymph Protein Concentrations

In accordance with the total hemolymph protein concentrations determined with the Bradford assay, the following dilutions of *T. molitor* and *Z. morio* total hemolymph proteins for cell treatments were made with the appropriate cell culture medium [µg/mL]: 15.625, 31.25, 62.5, 125, 250, 500, 1000, 2000, 4000. Following cell incubation, 100 µL of cell culture medium was carefully removed from each well, after which 100 µL of each treatment was added in four replicates. Thus, the final concentrations of *T. molitor* and *Z. morio* total hemolymph proteins used as treatments for cell toxicity assays were as follows [µg/mL]: 7.8125, 15.625, 31.25, 62.5, 125, 250, 500, 1000, 2000. For control, 100 µL of appropriate cell culture medium was added per well. The plates were incubated for 24 h, 48 h, and 72 h at 37 °C and 5% CO_2_ in a humified CO_2_ incubator.

#### 4.4.3. MTT Assay

After 24 h, 48 h, and 72 h of incubation, an MTT assay (3-(4,5-dimethylthiazol-2-yl)-2,5-diphenyltetrazolium bromide) was performed to measure cellular metabolic activity, which is an indicator of cell viability, proliferation, and cytotoxic effects. The culture medium was carefully removed from treated and control wells and replaced with 100 µL/well of MTT (5 mg MTT/10 mL serum-free appropriate medium). The plates were incubated for 3 h at 37 °C and 5% CO_2_ in a humified CO_2_ incubator. After incubation, the solution was carefully removed from the wells and replaced with 100 µL/well of solvent (42 µL 35% HCl/10 mL isopropanol, i.e., 0.04 M HCl in isopropanol). The plates were incubated for 10 min at room temperature in the dark. After that, absorbance was measured at wavelengths of 540 nm (A_540_) and 690 nm (A_690_) using a Multiskan GO spectrophotometer and SkanIt version 5.0 software. This experiment was replicated independently three times for each incubation time point—24 h, 48 h, and 72 h. The obtained A_690_ values for each well were subtracted from the A_540_ values:

A_sample_ = A_540_ − A_690_,

after which the average value of control sample absorbance (A_control_) was determined, and the percentage of cell viability (% cell viability) was calculated according to the following formula:

% cell viability = A_sample_ × 100/A_control_

### 4.5. Statistical Data Analysis—One-Way and Two-Way ANOVA

The results of the biochemical analyses and cytotoxicity assays were statistically analyzed using the Statistica version 14.0 software (StatSoft, Inc., Tulsa, OK, USA). First, normal distribution and equal group variance of the data were confirmed with the Brown–Forsythe test. Next, the statistical significance of the differences was tested with a one-way analysis of variance (one-way ANOVA) followed by Tukey’s post hoc analysis for a significance level of *p* < 0.05. Additionally, the combined effects of hemolymph protein concentration and incubation time, as well as hemolymph protein concentration and insect species at different time points, on cell viability, were tested using two-way analysis of variance (two-way ANOVA) followed by Tukey’s post hoc analysis for a significance level of *p* < 0.05. The results are presented as bar charts, and graphs were created using GraphPad Prism version 8.0.2 software (Dotmatics, Boston, MA, USA). Statistically significant results are highlighted with asterisks (explanation given in individual graph descriptions).

## 5. Conclusions

In this study, it was shown that the *T. molitor* larval hemolymph is rich in proteins and total carbohydrates, and it also exhibits a lower level of lipid peroxidation and antioxidant capacity than larval *Z. morio* hemolymph. Additionally, *T. molitor* larval hemolymph exerts a weaker cytotoxic effect on both cancer and normometabolic cell lines compared to hemolymph from *Z. morio* larvae. Specifically, the cytotoxic effect was observed only after treatment with the highest concentrations of hemolymph proteins, and it was generally more potent on cancer cells. Considering that the hemolymph of *T. molitor* larvae was found to be richer in carbohydrates compared to *Z. morio* larval hemolymph, this could explain its milder cytotoxic effects. The higher carbohydrate content could translate into more metabolic fuel for cell proliferation after the treatments. These findings align well with the status of *T. molitor* as an edible species and an alternative source of proteins to be used in human and animal consumption.

Regarding the hemolymph of *Z. morio*, it was demonstrated to contain high concentrations of total proteins, a high level of lipid peroxidation, and therefore high antioxidative capacity, and a low level of total carbohydrates in comparison to *T. molitor* larval hemolymph. These characteristics are potentially linked to its more potent cytotoxic activity against both cancer and normometabolic cells compared to *T. molitor* hemolymph. However, considering that *Z. morio* hemolymph exhibited greater cytotoxicity to cancer cells, it is hypothesized to contain anticancer agents warranting further investigation. Concerning the exploration of *Z. morio* hemolymph’s potential in a food-related context, its documented high antioxidant capacity suggests the presence of antioxidant molecules that could potentially enhance food quality, substituting synthetic ones commonly utilized. Therefore, further research into the hemolymph of both insect species from this study, as well as specific constituents of the hemolymph, is necessary to fully explore their potential for application in various fields of the food, feed, and pharmaceutical industries. Indeed, following the results of this study, the next step would be to ascertain the specific biomolecular composition of the hemolymph from the larvae of these insects, which would help identify key bioagents responsible for the cytotoxic effects recorded here. Additionally, while different degrees of hemolymph cytotoxic effects on both analyzed cell lines were shown, further analyses must be undertaken to better describe them and show whether these effects are due to some inherent anticancer properties of these hemolymph extracts, or due to some other antiproliferative mechanisms.

## Data Availability

The data presented in this study are available from the corresponding authors upon reasonable request.
